# A Multipulse Radar Signal Recognition Approach via HRF-Net Deep Learning Models

**DOI:** 10.1155/2021/9955130

**Published:** 2021-06-02

**Authors:** Ji Li, Huiqiang Zhang, Jianping Ou, Wei Wang

**Affiliations:** ^1^School of Computer and Communication Engineering, Changsha University of Science and Technology, Changsha 410114, China; ^2^ATR Key Lab, National University of Defense Technology, Changsha 410073, China

## Abstract

In the field of electronic countermeasure, the recognition of radar signals is extremely important. This paper uses GNU Radio and Universal Software Radio Peripherals to generate 10 classes of close-to-real multipulse radar signals, namely, Barker, Chaotic, EQFM, Frank, FSK, LFM, LOFM, OFDM, P1, and P2. In order to obtain the time-frequency image (TFI) of the multipulse radar signal, the signal is Choi–Williams distribution (CWD) transformed. Aiming at the features of the multipulse radar signal TFI, we designed a distinguishing feature fusion extraction module (DFFE) and proposed a new HRF-Net deep learning model based on this module. The model has relatively few parameters and calculations. The experiments were carried out at the signal-to-noise ratio (SNR) of −14 ∼ 4 dB. In the case of −6 dB, the recognition result of HRF-Net reached 99.583% and the recognition result of the network still reached 97.500% under −14 dB. Compared with other methods, HRF-Nets have relatively better generalization and robustness.

## 1. Introduction

The external electromagnetic environment is becoming more and more complex, which brings severe challenges to electronic reconnaissance and electronic countermeasures systems. In the process of electronic countermeasures, the rapid and accurate identification of intercepted signals can give priority to the right to control information. However, the intercepted enemy signal is not only a single pulse signal, but also a multipulse signal, so the identification of multipulse is also extremely important.

Traditional radar signal recognition technology usually uses pulse description words (PDW) to match conventional parameters and designs feature extraction algorithms and classifiers for recognition. Wenqiang Zhang et al. [1] designed a TPOT-LIME algorithm, which can recognize radar signals from multiple aspects. Krzysztof Konopko et al. [2] used Wigner–Ville distribution to perform time-frequency analysis on the signal, then used a probability density function estimator to extract feature vectors, and finally used a statistical classifier to recognize radar signals. The recognition accuracy is better, but the recognized signal classes are less. Jian Guo et al. [3] designed an FCBF-AdaBoost algorithm to identify radar signals and achieved good results. Qiang Guo et al. [4] designed a method that combines the main ridge slice and cloud model and constructed a feature vector for radar signal recognition, which has a high recognition rate. Jingchao Li and Ying [5] designed an entropy feature algorithm. The algorithm described the distribution features of different classes of radar signals by extracting odd-spectrum Shannon entropy and odd-spectrum exponential entropy features and had a higher recognition rate under low SNR.

However, with the increasingly serious external interference, the signal features are easily submerged by external interference. The traditional radar signal recognition method also needs to carry out complex feature design, which is difficult to achieve high recognition results. With the development of deep learning, Convolutional Neural Networks (CNNs) have been widely used. The network is widely used in image classification, semantic segmentation, target detection, and other directions. Muqing Zhang et al. [6] designed an algorithm based on stacked autoencoders and support vector machines (SVM). This method obtained the time-frequency diagram of radar signals through Choi–Williams distribution, then used stacked autoencoders to automatically extract features, and finally completed signal recognition through SVM. Shunjun Wei et al. [7] designed a new type of network combining shallow CNN, LSTM, and deep DNN, which has a good recognition effect on a variety of radar signals. Li ji et al. [8] proposed an IIF-Net deep learning model, which achieved good recognition effect under low SNR. Guo, Limin et al. [9] designed an improved AlexNet network, and through time-frequency analysis of the signal, the overall recognition rate is higher under low SNR. Yihan Xiao et al. [10] designed a feature fusion algorithm, combined with an improved CNN, and got better recognition results.

In this paper, GNU Radio, USRP N210, and USRP-LW N210 are used to generate close-to-real radar signals with high reliability. 10 classes of multipulse radar signals are generated between −14 and 4 dB in SNR, namely, Barker, Chaotic, EQFM, Frank, FSK, LFM, LOFM, OFDM, P1, and P2. Various signals through the CWD are used to generate two-dimensional TFIs. Different radar signals TFI have larger repetitive similar regions, while the distinctive feature regions are relatively small. In order to solve the above difficult, this paper designs a distinguishing feature fusion extraction module (DFFE) and proposes a new high-resolution feature fusion extraction network (HRF-Net) based on this module.

## 2. DFFE Module and HRF-Nets

### 2.1. CNNS

**Figure 1:**
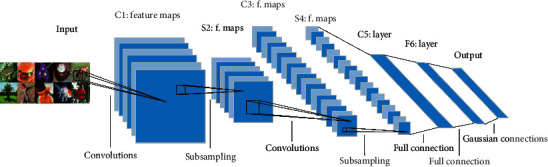
The basic architecture of CNN.

CNNS can extract target features adaptively.  Figure 1 shows its network architecture [11]. Wang Wei et al. [12] gave a detailed analysis and introduction to CNN. In semantic segmentation, CNN can extract image features and achieve image pixel-level classification [13]. In order to improve the recognition effect, the image can be preprocessed, such as superresolution reconstruction [14], and attention mechanism can also be introduced to improve the performance of the network [15]. AlexNet [16] applies ReLU, LRN [17], and Dropout [18] at the same time. Simonyan and Zisserman [19] proposed the 3×3 small convolution filter in visual geometry group networks (VGGNets), and the network reached 19 layers. But when the network has been trained enough, the performance of the network will decrease instead. Residual net (ResNet) [20] used skip connections to solve this problem and continued to increase the depth of the network.

Affected by the ResNet, Huang, Gao et al. [21] designed a dense connection mechanism that can connect all layers to each other and achieved reuse feature. Wang et al. combined DenseNet and MobileNet [22] to design a Dense-MobileNet [23], which achieved higher recognition rate and reduced the amount of network parameters and calculations.

### 2.2. DFFE Module

**Figure 2:**
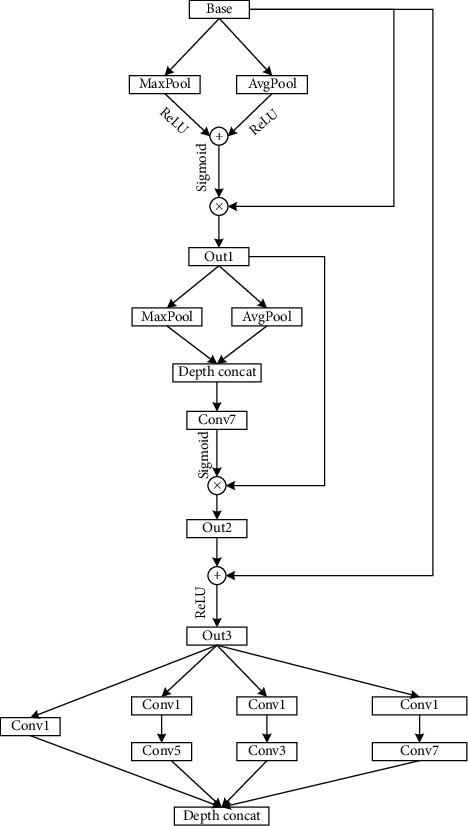
Structure of DFFE.

The TFIs of different radar signals have larger repeating similar regions, and the distinguishing feature regions are smaller. Therefore, this paper designs a distinguishing feature fusion extraction module (DFFE).  Figure 2 shows its structure.

First, in the spatial dimension, MaxPool and AvgPool are simultaneously performed on the input feature map. The feature map is compressed in the spatial dimension to obtain two one-dimensional vectors, which, respectively, represent the channel weight coefficients of the two feature maps. Then, through the ReLU activation function, the two channel weight coefficients are added together. A comprehensive analysis is performed to highlight the highly correlated channels, suppress the irrelevant channels, and focus on which input channels are more distinguishable. Then, we use the Sigmoid activation function. Finally, the one-dimensional channel weight vector is multiplied by the input feature map, keeping the input size unchanged, so the channel weight feature map Out1 is obtained.

In the channel dimension, MaxPool and AvgPool are performed on Out1. The channel dimension is compressed to obtain two two-dimensional spatial weight feature matrices, which are stitched together according to the channel dimension. A feature map with 2 channels is obtained. After that, Conv7 is used for convolution operation. Then, Sigmoid function is used for activation to obtain a comprehensive two-dimensional spatial weight feature matrix. It emphasizes the spatial position of high correlation and weakens the spatial position of less correlation, focusing on which areas of the input image are more distinguishable. Finally, the obtained two-dimensional feature matrix is multiplied with Out1, keeping the input size unchanged, so the feature map Out2, which is more distinguishable in space and channel, is obtained.

As the network deepens, problems such as image information loss and network degradation will occur. Adding skip connections can solve these problems. Therefore, we add the obtained feature map Out2 to the original input feature map “Base.” And after the activation function ReLU, we obtain the feature map Out3 with complete information and more spatial and channel resolution. Next, we perform multiscale feature extraction on Out3 by using “Conv7,″ “Conv5,″ “Conv3,″ and “Conv1,″ respectively. Larger convolution kernels have larger receptive fields and strong semantic information representation ability. Smaller convolution kernels have smaller receptive fields, strong geometric detail information representation ability, and high resolution. Therefore, a variety of sizes of convolution kernels are used to perform feature fusion extraction on Out3, so the high-resolution features are obtained.

### 2.3. HRF-Nets

Based on the DFFE module, we propose three deep convolutional neural network structures, namely, high-resolution feature fusion extraction networks (HRF-Nets), which are HRF-Net157, HRF-Net187, and HRF-Net217. Among them, C-[MaxPool, AvgPool] represents the compression of the image spatial dimensions to obtain a feature map with channel weight coefficients. S-[MaxPool, AvgPool] means compressing the image channel dimensions to obtain a feature map with spatial weight coefficients.  Table 1 is the network structure.

The field of radar electronic countermeasures has high requirements for time delay, so the network needs smaller computational costs. Many classic CNNs use three-layer fully connected layers such as AlexNet and VGGNets, which have a high computational cost and take a long time. Therefore, this paper first adopts the Global Average Pooling (GAP) [24] and then uses single-layer full connection, which obviously reduces the calculation cost and makes the network have relatively high real-time performance.

### 2.4. Network Complexity

The classifier occupies a large part of the calculation and parameter amount in the network, and the difference of the classifier greatly affects the performance and calculation cost of the network. In this paper, 10 classes of multipulse radar signals are recognized. Assuming that the output of the last layer of the network is *H* × *W* × *D*. The parameter amount of using three-layer full connection as the classifier is 16,818,184+4096 × *H* × *W* × *D*, the parameter amount of using single-layer full connection is *H* × *W* × *D* × 10+10, and the parameter amount of using GAP is  *D*+D × 10+10.

**Figure 3:**
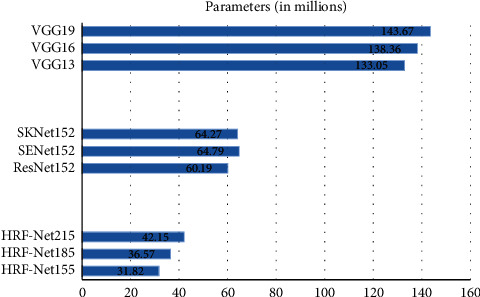
Parameters for different networks.

**Figure 4:**
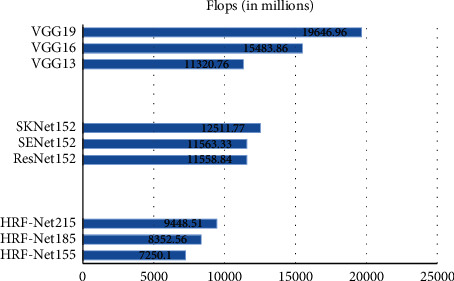
FLOPs.

Figure 3 shows the amount of parameters for different networks, and Figure 4 shows the amount of calculation.

According to Figure 3, it can be seen that when the network depth gradually increases, the amount of network parameters of the same type is gradually increasing, indicating that the network depth affects the size of the parameter amount to a certain extent. The three-layer fully connected layer is the classifier of VGGNets. The HRF-Nets classifier uses GAP plus a single-layer fully connected. Although the VGG13 network has only 13 layers, its parameter quantity is 4.18 times that of HRF-Net157, 3.64 times that of HRF-Net187, and 3.16 times that of HRF-Net217. Therefore, the classifier is a key factor affecting the amount of network parameters. The parameters of SKNet152, SEnet152, and ResNet152 are larger than those of HRF-Net157. The parameter of ResNet152 is 1.89 times that of HRF-Net157, which has more parameters of about 28.37 million.

According to Figure 4, it can be seen that the VGGNets have a huge amount of calculation. The 13-layer VGG network has a floating point calculation amount of 11.321 billion, which is 1.56 times that of the 157-layer HRF-Net. Compared with the ResNet152, HRF-Net157 has a deeper depth. But the calculation of ResNet152 is 1.59 times that of HRF-Net157, which has an increase of 4.309 billion. This is because ResNet152 uses a large number of convolutional layers. Without considering the bias, the calculation amount of the convolutional layer is (2*∗C*_*int*_*∗K*^2^ − 1)*∗C*_out_*∗H*_out_*∗W*_out_. HRF-Nets have many pooling layers, and the calculation amount of the pooling layer is *K*^2^*∗C*_out_*∗H*_out_*∗W*_out_. Therefore, HRF-Net157 has a smaller amount of calculation than ResNet152. The calculation amount of HRF-Net217 is 30.32% more than that of HRF-Net157, and the calculation amount of HRF-Net187 is 15.21% more than that of HRF-Net157. Radar electronic countermeasure system requires low delay, especially in small devices such as missiles. The memory is insufficient, and the hardware conditions do not support too many parameters and calculations. The HRF-Net157 is relatively small in terms of parameters and calculations. Therefore, when the recognition result of the signal differs slightly, HRF-Net157 has the highest cost performance and is a better choice.

## 3. Experimental Results

### 3.1. Dataset

In this paper, the multipulse radar signal is generated by GNU Radio USRP N210, and USRP-LW N210. When we intercept the enemy signal, our interception should be a multipulse signal. Therefore, this paper generates a multipulse radar signal with 4 pulses. Because of the different distribution of noise, the signals between radar pulses are not exactly the same. In order to obtain the TFI of the radar signal, the signal is CWD transformed. Different from SAR image [25] and high-resolution radar target image [26], TFI has high definition, which is good for signal recognition.

Different time-frequency analysis algorithms have different characteristics. Among them, Gabor transform localizes time and frequency at the same time, which can better describe the transient structure in the signal. Its time-frequency resolution is completely determined by the Gaussian window. The Wegener–Wiley distribution (WVD) is to distribute the energy of the signal in the time-frequency plane. It has a good time-frequency focus. Affected by the interference of the cross term, its various smoothing improvement methods can reduce the cross-term interference but reduces the time-frequency focus.

**Figure 5:**
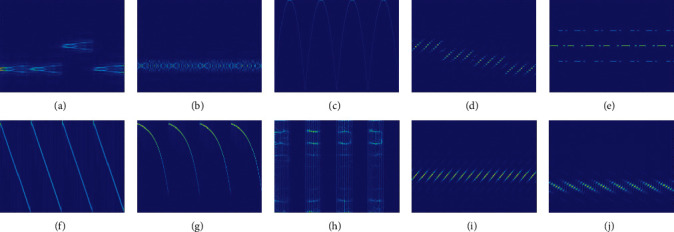
TFIs of 10 multipulse radar signals. (a) Barker, (b) Chaotic, (c) EQFM, (d) Frank, (e) FSK, (f) LFM, (g) LOFM, (h) OFDM, (i) P1, and (j) P2.

In order to improve the recognition rate of the signal, we need high-resolution images. The CWD has the characteristics of minimal cross-term interference and has high definition and resolution for different signals. The radar signal data set in this paper contains 10 classes of signals. There are 2880 TFIs for each class of signal. We add Gaussian white noise to the signal. In the SNR of −14 ∼ 4 dB, there are a total of 28800 samples, including 21,600 in the training set and 7,200 in the test set.  Figure 5 is the TFI after the signal passes through the CWD.

According to Figure 5, it can be seen that different radar signals TFI have a large number of repeated similar regions, while the regions of distinguishing features are relatively small. Therefore, this paper designs a DFFE module, which can focus on extracting regional features with strong resolution and achieve the purpose of improving the signal recognition rate.

### 3.2. Preprocessing and Experiment Setup

In the process of data preprocessing, we downsample the image, and the resolution is fixed to 224^*∗*^224. Then, the data is expanded, and the image is randomly flipped horizontally, vertically flipped randomly, and rotated 90° randomly. Data expansion increases the complexity of the image and improves the performance of the network. USRP N210 and USRP-LW N210 are hardware devices for signal generation. Among them, its ADC Sampling Rate is 100MS/s, DAC Sampling Rate is 400MS/s, and LO accuracy is 2.5 ppm.

In the experiment, we set some parameters. Among them, the bandwidth of the signal is 4MHZ, the batch size is 16, the initial learning rate is 0.001, the weight decay is 5e-4, and the momentum is 0.9. The experiment is conducted for a total of 60 cycles, and the final results are the average of the last 10 cycles. The training and testing process of the network is implemented on the server. Among them, we use the PyTorch framework. Its operating system is Ubuntu 14.04.5 LTS, GPU is GeForce GTX TITAN *X*, and CUDA is CUDA 8.0.61.

### 3.3. Experimental Results

**Figure 6:**
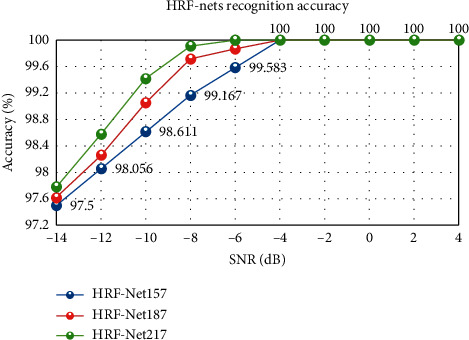
HRF-Nets recognition accuracy at different depths.

In this paper, we add noise to the signal to keep the SNR at −14 ∼ 4 dB. Then, we generate more realistic multipulse radar signals through GNU Radio and USRP N210, USRP-LW N210. We use HRF-Net with different depths to identify multipulse radar signals.  Figure 6 shows the experimental results.

According to Figure 6, when HRF-Nets have a SNR of -8 dB, the network recognition results are all over 99%. When HRF-Nets are at a SNR of -14 dB, noise interference has already had a great impact on the signal. However, the recognition result of the network still exceeds 97%, which shows that the HRF-Nets network has good robustness. The recognition rate of HRF-Net157 is slightly lower (within 1%) than that of the other two networks. It indicates that as the network deepens, its signal feature extraction ability has approached saturation, and the signal recognition rate has not improved significantly. HRF-Net217 and HRF-Net187 have more parameters by 32.46% and 14.93% than HRF-Net157, and more calculations by 30.32% and 15.21% than HRF-Net157. It can be seen from Figure 6 that the recognition result of HRF-Net217 is the best. Compared with HRF-Net157, the recognition rate is improved by no more than 1%, but the computational cost of the network has increased obviously. Through comprehensive analysis, we believe that HRF-Net157 has the highest cost performance. We also compare HRF-Net157 with other CNN networks, and Table 2 shows the recognition effect.

According to Table 2, the recognition performance of HRF-Nets is relatively high when the SNR is between -14 and 4 dB. As the electromagnetic environment of the modern battlefield is becoming more and more complex, the signal interference is increasing. The recognition of radar signals under low SNR is of greater significance, and its recognition is more difficult. In the case of −14 dB, the recognition result of HRF-Net157 is about 7% higher than that of VGGNets, and the calculation cost of VGGNets is too high, and the delay is long. Therefore, the VGGNets cannot be applied to the field of low delay radar electronic countermeasures.

In the recognition results of 10 multipulse radars, HRF-Net157 is about 2% higher than ResNet152, SEnet152, and SKNet152. It is 2.418% higher than ResNet152, while the calculation and parameter amount of HRF-Net157 are relatively small. Although ResNet152 uses skip connections, it maintains information integrity. However, the TFI distinguishing feature area of the multipulse radar signal is small, and the repetitive area is large. When ResNet152 extracts image features, it performs the same processing on the image globally and has no specificity to the distinctive feature area. The DFFE module focuses on extracting high-resolution regional features of the image, improves the recognition effect of the network, and enhances generalization.

Table 3 shows the comparison result of HRF-Net157 with other methods, and it can be seen that the CLDNN network has a better recognition result at a SNR of over −8 dB, reaching more than 90%, but when SNR is within the range of −14 ∼ −8 dB, its recognition rate is relatively poor. HRF-Net157 has a better recognition rate at −14 dB. The comprehensive recognition rate of HRF-Net157 is still as high as 97.5%, indicating that HRF-Net157 can still fully extract image features under low SNR, so it has strong anti-interference ability and good robustness. FCBF-AdaBoost adopts traditional feature selection and classifier design, which has a good recognition rate under the condition of less interference. But it is mostly for a certain class of image recognition. In a multitask and low SNR environment, its recognition rate is relatively poor. CNN-KCRDP, AlexNet, and I–CNN all combine deep learning to recognize images to a certain extent and can adaptively extract image features. Their recognition rates are not much different from HRF-Net157 when SNR is above −6 dB. But, in the case of more serious interference, the signal features are submerged by noise. HRF-Net157 can extract more distinguishable features to a greater extent through the DFFE module. Therefore, when the interference is large, a better recognition effect can still be achieved.

Table 4 shows the recognition results of HRF-Nets for different signals. In the case of -14 dB, the difference between the recognition results of the same class of radar signal for the three depths of HRF-Net is only about 2%. Deepening the network increases a lot of parameters and calculations, but the recognition effect is not significantly improved. Among the 10 multipulse radar signals, the recognition results of Barker, Chaotic, Frank, OFDM, FSK, LFM, EQFM, and LOFM in HRF-Nets all reached more than 94%. The recognition effects of P1 and P2 are relatively poor, around 90%, and the fluctuation is large. We choose HRF-Net157 with the best cost performance and generate a confusion matrix at −14 dB for further analysis.

**Figure 7:**
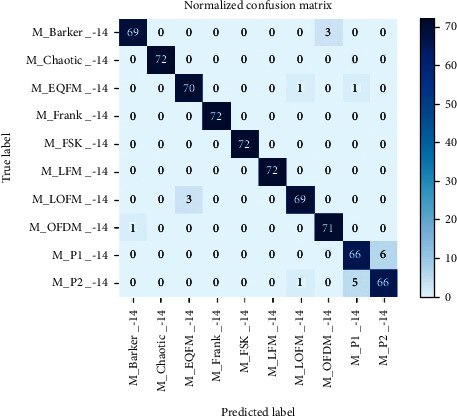
Confusion matrix of HRF-Net157(-14 dB).

According to Figure 7, it can be seen that the classes of errors identified in P1 are all P2. Among the 6 errors of P2, 5 are P1. According to Figure 5, it can be seen that the TFI of P1 and P2 has a certain degree of similarity. In the case of −14 dB, the interference of noise has largely covered the features of the image, making the similarity of P1 and P2 increase, and further improved the difficulty of identification. However, the recognition rate of P1 and P2 still reaches about 90%. The HRF-Nets proposed in this paper can focus on extracting high-resolution image features for images with small distinguishing regions and obtain better recognition results. The comprehensive recognition rate of HRF-Net157 under -14 dB reached 97.500%.

### 3.4. Experiment Analysis

In this paper, three depths of HRF-Net are proposed, namely, HRF-Net157, HRF-Net187, and HRF-Net217. According to the experimental results, the recognition rate of the signal is more than 99% when SNR is above -6 dB. In the case of −14 dB, the recognition results of the network also reached 97.500%. With the increase of network depth, the difference in recognition rate of HRF-Net157, HRF-Net187, and HRF-Net217 is only within 1%, but the computational cost has increased obviously. Taking into account comprehensive considerations, we believe that HRF-Net157 is the most cost-effective. In the process of comparing with other CNNs, it is found that the recognition rate of HRF-Net157 between −14 dB and −6 dB is higher than other CNNs, which is more obvious in the case of low SNR. When comparing with other methods, it is found that the recognition results of HRF-Net157 are better than other methods under the condition of −14 dB. When compared with other methods, it is found that the signal recognition rate of HRF-Net157 is higher than other methods under the condition of −14 dB. It has better robustness. In the case of −14 dB, HRF-Nets also have a good recognition effect on different classes of radar signals.

According to the TFI of the multipulse radar signal, we can see that the similarity area between different images is larger, and the distinguishing area is smaller. Therefore, when extracting image features, the importance of different areas of the image should be considered, focusing on extracting more distinguishable regional features. The network depth should be kept moderate. The image features cannot be fully extracted if the network is too shallow. The recognition rates of networks have not changed significantly when the network is too deep. There may also be network degradation problems, and the amount of parameters and calculations will increase significantly. The use of skip connections can maintain the integrity of image information. The classifier uses GAP followed by a single-layer full connection, which can greatly reduce the computational cost of the network. The DFFE module designed in this paper can perform distinguishing feature fusion extraction of images. First, Maxpool and Avgpool are used to compress the spatial dimensions to obtain feature maps with channel weights. Then, Maxpool and Avgpool are used to compress the channel dimensions, and the spatial weight feature map is obtained. Finally, the features of images are extracted by multiscale fusion through Conv1, Conv3, Conv5, and Conv7 to obtain high-resolution features and improve the signal recognition rate.

## 4. Conclusions

In this paper, we use GNU Radio, USRP N210, and USRP-LW N210 to generate close-to-real multipulse radar signals and then perform CWD transformation on the echo signal to get TFI. Aiming at the features of the multipulse radar signal TFI, a DFFE module is designed, which can perform distinguishing fusion extraction of image features. Based on the DFFE module, we proposed three deep CNN structures, that is, HRF-Net157, HRF-Net187, and HRF-Net217. The nets can identify 10 classes of radar signals and have good generalization. Through comprehensive comparison, we believe that HRF-Net157 is the most cost-effective. In the case of a SNR of −14 dB, there is still a recognition rate of 97.500%, with better robustness and lower computational cost. In radar systems that require low delay, HRF-Nets have certain advantages and can be further studied in the areas of radar interference recognition and radar radiation source recognition.

## Figures and Tables

**Table 1:** HRF-Nets configurations.

HRF-Net157		HRF-Net187		HRF-Net217	
Conv7-64, stride:2					
3×3Maxpool, stride:2					

Conv1-64	×3	Conv1-64	×3	Conv1-64	×3
Conv3-64		Conv3-64		Conv3-64	
C-[MaxPool, AvgPool]		C-[MaxPool, AvgPool]		C-[MaxPool, AvgPool]	
S-[MaxPool, AvgPool]		S-[MaxPool, AvgPool]		S-[MaxPool, AvgPool]	
Conv7-64		Conv7-64		Conv7-64	
Conv1-256		Conv1-256		Conv1-256	

*DFFE-256*					
Conv1-128	×7	Conv1-128	×8	Conv1-128	×8
Conv3-128		Conv3-128		Conv3-128	
C-[MaxPool, AvgPool]		C-[MaxPool, AvgPool]		C-[MaxPool, AvgPool]	
S-[MaxPool, AvgPool]		S-[MaxPool, AvgPool]		S-[MaxPool, AvgPool]	
Conv7-128		Conv7-128		Conv7-128	
Conv1-512		Conv1-512		Conv1-512	

*DFFE-512*					
Conv1-256	×10	Conv1-256	×14	Conv1-256	×19
Conv3-256		Conv3-256		Conv3-256	
C-[MaxPool, AvgPool]		C-[MaxPool, AvgPool]		C-[MaxPool, AvgPool]	
S-[MaxPool, AvgPool]		S-[MaxPool, AvgPool]		S-[MaxPool, AvgPool]	
Conv7-256		Conv7-256		Conv7-256	
Conv1-1024		Conv1-1024		Conv1-1024	

*DFFE-1024*					
Conv1-512	×3	Conv1-512	×3	Conv1-512	×3
Conv3-512		Conv3-512		Conv3-512	
C-[MaxPool, AvgPool]		C-[MaxPool, AvgPool]		C-[MaxPool, AvgPool]	
S-[MaxPool, AvgPool]		S-[MaxPool, AvgPool]		S-[MaxPool, AvgPool]	
Conv7-512		Conv7-512		Conv7-512	
Conv1-2048		Conv1-2048		Conv1-2048	
Classifier, Softmax					

**Table 2:** Recognition results of different networks (%).

SNR (dB)	ResNet152	SENet152	SKNet152	VGG13	VGG16	VGG19	HRF-Net157
−14	95.082	95.253	95.535	89.268	90.366	90.851	97.500
−12	96.374	96.862	97.134	91.423	93.514	93.735	98.056
−10	97.746	98.254	98.481	93.526	94.316	95.242	98.611
−8	98.356	98.426	98.768	95.628	96.211	97.522	99.167
−6	99.161	99.287	99.442	98.254	98.856	99.082	99.583
−4	100	100	100	99.142	99.627	99.855	100
−2	100	100	100	100	100	100	100
0	100	100	100	100	100	100	100
2	100	100	100	100	100	100	100
4	100	100	100	100	100	100	100

**Table 3:** Recognition results of other methods (%).

Methods	−14	−12	−10	−8	−6	−4	−2	0	2	4
CLDNN [7]	46	66	83	92	97	98	99	100	100	100
CNN-KCRDP [27]	—	—	88	94	97	98	100	100	100	100
AlexNet [9]	—	—	82	89	92	93	96	99	100	100
I-CNN [28]	—	—	55	80	96.10	—	100	100	100	100
FCBF-AdaBoost [3]	—	—	—	—	—	—	—	94.46	96.86	98.75
HRF-Net157	97.500	98.056	98.611	99.167	99.583	100	100	100	100	100

**Table 4:** HRF-Nets recognition results of different signals (−14 dB) (%).

Signal	HRF-Net157	HRF-Net187	HRF-Net217
Barker	98.611	100	100
Chaotic	100	100	100
EQFM	97.222	100	98.241
Frank	100	97.536	100
FSK	100	100	100
LFM	100	100	100
LOFM	94.444	96.538	96.524
OFDM	100	98.564	100
P1	87.500	89.536	89.422
P2	93.056	92.467	91.362

## Data Availability

The data source is available from the author at hqzhang9013@163.com.
